# Seroprevalence of pertussis antitoxin (anti-PT) in Sweden before and 10 years after the introduction of a universal childhood pertussis vaccination program

**DOI:** 10.1111/j.1600-0463.2009.02554.x

**Published:** 2009-12

**Authors:** Hans O Hallander, Mikael Andersson, Lennart Gustafsson, Margaretha Ljungman, Eva Netterlid

**Affiliations:** Swedish Institute for Infectious Disease ControlSolna, Sweden

**Keywords:** Pertussis, ELISA IgG anti-PT, seroepidemiology, Sweden

## Abstract

The prevalence of IgG ELISA antibodies against pertussis toxin (anti-PT) was studied in two Swedish seroepidemiological studies. One was performed in 1997 when the new pertussis vaccination program was 1 year old (n = 3420). In 2007, when Pa vaccines had been used countrywide for 10 years in the universal child vaccination program, this study was repeated to analyze the effect of vaccination on anti-PT prevalence (n = 2379). Before the statistical analysis of seroprevalence, children vaccinated within the last 2 years before the serosurveys were excluded. The results indicate a reduced exposure to *Bordetella pertussis* in the population. The proportion of sera without measurable anti-PT antibodies increased significantly, aggregated over all comparable age groups, from 3.8% in people sampled in 1997 to 16.3% in people sampled in 2007. For cord blood, 1% was without measurable anti-PT antibodies in 1997 compared to a significantly higher level, 12%, in 2007. With anti-PT concentrations of ≥50 and ≥100 EU/ml as cutoff points for ‘recent infection’ the proportion above the cutoff points for younger children was significantly higher in 1997 than in 2007 at both cutoff points. For all adults, 20 years of age and older, the difference in proportions above the lower cutoff point was close to statistically significant, comparing 1997 with 2007. This was not the case at 100 EU/ml. In the 1997 samples of children, there was a significant downward trend of ‘recent infections’ at both cutoff points for three sampled age groups between 5 and 15 years of age from 21% at 5.0–5.5 years of age to 7% at 14.7–15.7 years for the lowest cutoff. In the 2007 samples of children, on the contrary, there was a significant continuous upward trend of ‘recent infections’, at both cutoff points, for four sampled age groups between 4 and 18 years of age – from 4% at 4–5 years of age to 16% at 17–18 years at the lowest cutoff. The continuous increase, with age of children with high anti-PT concentrations, supports the recent change in the general Swedish childhood vaccination program to include a pre-school booster at 5–6 years and a school-leaving booster at 14–16 years of age.

Although vaccination against pertussis has been very effective in terms of reduced incidences of reported disease, pertussis continues to be a health problem in Sweden as well as in other countries (1, 2). In particular, young unvaccinated or partially vaccinated infants are at risk of serious disease. Sixteen European countries provided national surveillance data of pertussis for the period 1998–2002. Among children younger than 1 year, 70% of cases with a pertussis disease were admitted to hospital (3). Adolescents and adults provide an often-undiagnosed reservoir for infections in this age group (4, 5).

The resurgence of pertussis in peaks every 3–5 years is most often explained as the result of waning immunity (3, 6–8). Among older children, adolescents and adults, the disease is often milder, probably because of earlier vaccination or infection. It is also widely recognized that routine notifications/reports of pertussis disease underestimate the incidence of pertussis disease in all age groups, in particular, in the older ones (3, 9). Cherry (9), reviewing reports from the United States and Germany, estimated that rates of notified pertussis are 40–160 times lower than actual illness rates based on serology and that asymptomatic infections are 4–22 times more common than symptomatic infections. It may be that vaccination protects better against disease than against infection and transmission. To what extent transmission of *Bordetella pertussis* is reduced by vaccination has, however, been a matter of discussion. There is evidence that pertussis vaccination is highly effective in reducing transmission from vaccinated breakthrough cases (10, 11), but the role of vaccinated asymptomatic or mild cases in transmission remains to be established.

Production of pertussis toxin (PT) is unique for *B. pertussis* and the toxoid is used as one component in all acellular pertussis vaccines*.* Studies on prevalence of low anti-PT concentrations in different age groups have been used to target appropriate ages for booster vaccination (12–14), although the correlation of anti-PT with protection seems to be weak (15)*.* Anti-PT can, thus, be used in serosurveys as an indirect marker of antigenic pressure reflecting transmission of infection in the population. Such studies are of particular value if the studies can be repeated after significant changes, e.g. as in our case, after introduction of a new vaccination program.

In Sweden, the circumstances for studying pertussis vaccination efficacy and long-term effectiveness have been very favorable with one early period of DTPwc vaccination ending in 1979, one period of vaccination with acellular pertussis vaccines (Pa) starting in 1996 and no pertussis vaccination in the universal childhood vaccination program in between.

The reported incidences of laboratory-confirmed (i.e. culture or PCR positive) pertussis disease in Sweden has dropped 10-fold from 121–150 per 100 000 in 1994–1995 to 12–15 per 100 000, ten years later. Pertussis, however, constitutes the weakest part of the childhood vaccination program in Sweden. The highest incidence occurs in children below 5 months of age, most of them unvaccinated or vaccinated with one dose of a pertussis vaccine (1). It is evident that *B. pertussis* is still circulating also in Sweden, a country with consistently high vaccine coverage (98.5%).

The main aim of this study was to compare the ELISA IgG anti-PT sero-prevalence as well as the proportion of samples without measurable anti-PT concentrations (<1 EU/ml), in comparable age groups, between two Swedish seroepidemiological studies. Sero-prevalence is defined as the proportion of samples with an anti-PT concentration above a defined cutoff point. One such study was performed in 1997 when the new pertussis vaccination program had just started (16) and another in 2007, when Pa vaccines had been used countrywide for 10 years in the universal child vaccination program.

As anti-PT has not been correlated with protection, the focus in this study was on transmission of infection in the population. In two previous studies, we analyzed the amplitude and decay curves of anti-PT, first after vaccination with a 5-component pertussis vaccine (17) and then, after infection among both vaccinated and unvaccinated young children (18).

With knowledge about anti-PT peak values, decay and persistence of anti-PT antibodies after infection and after vaccination, we chose cutoff points for sero-prevalence. Knowledge about persistence was also used to exclude samples from age groups ‘recently’ vaccinated with a pertussis vaccine (within 2 years before the sero-sample was taken) to study the effect of 10 years of childhood vaccination, with acellular pertussis vaccines, on the antigenic anti-PT pressure in different age groups.

## Materials and methods

### Swedish seroepidemiological cross-sectional studies 1997 and 2007

*The Swedish vaccination program after 1996; primary immunization, early booster and vaccines*.

#### Primary immunization

Immunization against pertussis was reintroduced in the national childhood immunization schedule in 1996, after a 17-year interval without vaccination with a pertussis vaccine. Children born in Sweden 1996 and later are recommended three doses of acellular pertussis vaccine according to a 3–5–12 monthly schedule and the coverage rapidly reached a level of about 98.5%, a level shown to be more or less constant, since the reintroduction until today (1).

#### Boosters

In 2005, a revision of the national schedule was initiated. As a first step, a booster was recommended to children at approximately 10 years of age from the autumn 2005. The first cohort given this fourth dose of Pa were children born in 1995, i.e. the year before formal introduction of DTPa in infancy, because this cohort was, to a large extent, catch-up vaccinated before 2 years of age. The booster revision was changed in December 2006, and would, from 2007 onwards, include a fourth dose for children already at 5–6 years of age and a fifth dose for children at 14–16 years of age, starting with children born since 2002 (1, 19).

#### Vaccines in the universal childhood vaccination program

During the 10-year period after reintroduction of pertussis vaccination, the vaccines used in infancy differed in time and by geographical regions, but have all contained PT (1).

#### Seroepidemiological blood samples in studies in 1997 and 2007

In 1997 and 2007, cross-sectional seroepidemiological surveys were performed in Sweden. In both surveys, children and adults from the Swedish population were selected in randomized samples, stratified by age.

For the seroepidemiological analysis, all ‘recently’ vaccinated were excluded. As ‘recently’ vaccinated, we defined individuals with a pertussis vaccine dose within the last 2 years before the sero-sample was taken. Altogether, 125 samples from 1997 and 247 from 2007 were excluded for this reason. For details, see Table 1. For the statistical analysis of sero-prevalence, 3295 sera remained from not ‘recently’ vaccinated children and adults in 1997 and 2132 sera in 2007 (Table 1).

**Table 1:** Distribution by age in years in Swedish seroepidemiological materials from 1997 and 2007, divided into not recently vaccinated and those recently exposed to vaccination with a pertussis vaccine. Figures for ‘not recently vaccinated’ in bold represent the number of individuals used for comparison of sero-prevalence between 1997 and 2007

1997			2007		
	
Age group	Not recently vaccinated	Recently vaccinated	Age group	Not recently vaccinated	Recently vaccinated
2–2.5	30	69	2		26
			3	53	
**5–5.5**	**78**	56	**4–5**	**85**	33
			6–7	61	28
**8.7–9.7**	**238**		**8–9**	**122**	
10.7–11.7	254		10–11		131
			12–13	113	29
**14.7–15.7**	**251**		**14–15**	**161**	
			16	65	
**17.7–18.7**	**214**		**17–18**	**123**	
			19	49	
**20–34**	**732**	** **	**20–34**	**410**	
**35–49**	**485**	** **	**35–49**	**223**	
**50–64**	**521**	** **	**50–64**	**255**	
**65–**	**492**	** **	**65–**	**412**	
Total	3295	125	Total	2132	247

In 1997, 400 children per age group were randomly selected from the population register. The following six age groups of children were included 2.0–2.5, 5.0–5.5, 8.7–9.7, 10.7–11.7, 14.7–15.7 and 17.7–18.7 years. For these age groups, places, and nurses experienced in taking blood samples from young children were used.

The selection of adults was performed in a two-step procedure. First, a number of parishes in each county were randomly chosen. In the next step, individuals in each of the following four age groups were randomly selected from the population register: In age group 20–34, n = 1200 individuals, and in each of age groups 35–49, 50–64 and 65 years or older, 800 individuals were selected.

Altogether, 6000 individuals were randomly selected from the population register in the above-mentioned ten age groups. Blood samples were analyzed for ELISA IgG pertussis antitoxin from 3220 of planned 3600 individuals sampled in this way (16).

In addition, cord blood was taken from 200 newborn children, consecutively at three maternity hospitals, and analyzed for ELISA IgG pertussis antitoxin*.* The cord blood results were distributed into the age group of the mother (see also under Statistics). Thus, altogether 3420 blood samples were analyzed for IgG ant-PT antibodies in the 1997 seroepidemiological survey in ten age groups comparable with those in the 2007 sero-survey.

In 2007, 500 children from each of the two age groups 2–3 and 4–5 years of age, four hundred children from each of the five age groups, 6–7, 8–9, 10–11, 12–13, and 14–15 years of age, were randomly selected from the population register. The groups were based on the age as of March 31, 2007. For these age groups, places, and nurses experienced in taking blood samples from young children were used.

Adults and the oldest child groups, 800 individuals per group, in each of the following seven age groups were randomly selected from the population register ‘NAVET’: 16–17, 18–19, 20–34, 35–49, 50–64, 65–79, and 80 + years.

Altogether, 8600 individuals were randomly selected from the population register in the above-mentioned 14 age groups with the goal to reach about 4200 blood samples for analysis.

In addition, 400 cord blood samples were planned. Cord blood samples were taken consecutively at 10 randomized maternity hospitals. Before the statistical analysis, the cord blood results were distributed into the age group of the mother (see also under Statistics).

Blood samples were analyzed for ELISA IgG pertussis antitoxin for 2379 of planned 4600 individuals (the volume was too small in 59 cases). Of the analyzed samples, 377 were cord blood samples.

#### Comparison of the sero-surveys of 1997 and 2007

It was also necessary to construct as comparable age groups as possible in the two sero-surveys. Age groups sampled in 2007 were therefore analyzed and new ones were constructed to be as comparable as possible with age groups sampled in the 1997 survey.

To achieve this, the anti-PT concentrations in close age groups 2007 were compared by means of Wilcoxon test (see also under Statistics). In this process, also ‘unvaccinated’ children were excluded before the comparison, n = 284 in 1997 and n = 341 in 2007, since a comparable age group was missing. Age groups and figures in bold in Table 1 were used for comparison of sero-surveys of 1997 (n = 3011) and 2007 (n = 1791).

#### Serology, IgG ELISA for analysis of antibodies to pertussis toxin (anti-PT)

An indirect ELISA method was used at SMI to measure the concentrations of IgG antibodies to pertussis toxin. The general outlines have been described in previous publications (20, 21). The method was introduced in relation to Trial I and has not been changed since. The basic reagents are the same: Pertussis toxin (TOH15; SmithKline Beecham, Rixensart, Belgium) as used in previous studies (17, 22), diluted to 1 mg/l in phosphate buffered saline pH 7.4, was used for coating high binding polystyrene plates overnight at room temperature.

The results were internationally traceable to the FDA (Food and Drug Administration, USA)-reference human antiserum, HRP3, containing 200 EU/ml of IgG anti-PT, used as calibrator. A pool of fractioned human plasma (Ig42) containing 1040 EU/ml and diluted 1/20 was used as a monitor. The minimum level of detection (MLD) was 1 EU/ml. With defined limits for acceptance and adequate retesting, the within-day and between-day coefficients of variation were less than 15%. Before each new study, a panel of sera is tested to ensure traceability. The method is accredited and the laboratory participates in an external quality assessment program (23).

In the seroepidemiological studies, sera collected in 1997 were analyzed in 1999 and sera from 2007 in 2008. To achieve full comparability between 1997 and 2007, and for international traceability, a correction factor of +6.6% was introduced for 1997 measurement results and a correction factor of −7.9% for 2007.

### Ethics

A national ethical committee approved sample collection and analyses and the participants signed an informed consent for these procedures.

### Statistics

When constructing age groups in 2007 used for comparison with age groups in 1997, Wilcoxon tests were used to compare anti-PT levels in 1-year age groups to see if any significant differences could be observed. We tested 4-year olds against 5-year olds (p = 0.13), 8-year olds against 9-year olds (p = 0.25), 14-year olds against 15-year olds (p = 0.16), and 17-year olds against 18-year olds (p = 0.15). As none of these tests showed a significant difference, we aggregated these individuals in 2-year age groups to create as large age groups as possible from the 2007 year material. Hence, we considered the age groups 4–5, 8–9, 14–15 and 17–18 years in 2007 comparable to the age groups 5.0–5.5, 8.7–9.7, 14.7–15.7 and 17.7–18.7 in 1997 (see Table 1).

Wilcoxon tests were also used to compare cord blood results with results from blood samples from women of the same age groups as the mothers from whom the cord blood sample was taken. There were no significant differences. The p-values for 1997 and 2007 were 0.87 and 0.42, respectively. The mean anti-PT concentration for cord blood was 21.6 EU/ml in 1997 and 19.8 EU/ml in 2007. Corresponding concentrations for the control groups of women were 19.4 EU/ml, and 19.0 EU/ml.

When proportions of individuals with anti-PT above ≥50 EU/ml, and ≥100 EU/ml in 1997 against 2007 were compared, two-sided chi-squared tests were used separately on age groups 4–9, 14–18, and 20+ years. Similar tests were carried out on single age groups to compare proportions of anti-PT without measurable concentrations (<1 EU/ml). We also carried out chi-squared-trend tests on age groups 4–18 years and ≥20 years in 1997 and 2007 to investigate the possible age effects in anti-PT. A p-value below 0.05 was considered statistically significant.

The background to choose ≥50 and ≥100 EU/ml as cutoff points for sero-prevalence is described in a separate paper also commented under discussion (18).

## Results

### Anti-PT concentrations in ‘not recently vaccinated’ in 1997 and 2007

#### Allover anti-PT concentrations

**Fig. 1:**
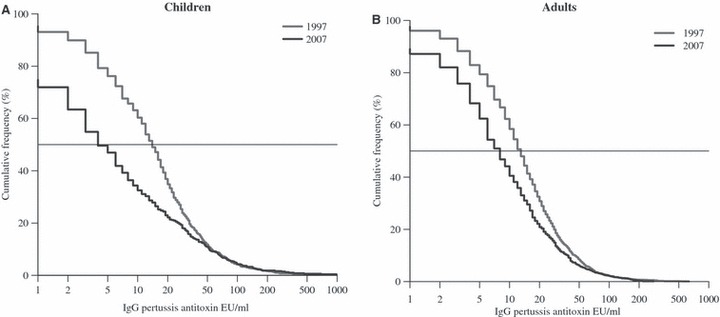
(A, B) Seroimmunity 1997 and 2007. Reverse cumulative distribution curves of ELISA IgG anti-PT antibodies in ‘not recently vaccinated’ children and adults in comparable age groups, all ages together.

The whole material of not ‘recently’ vaccinated is first used to illustrate the changes in antigenic pressure between 1997 and 2007, for children (Fig. 1A) and adults (Fig. 1B).

The highest reduction of anti-PT concentrations in 2007 compared to 1997 is seen in children. The median value for children fell from about 13.9 EU/ml in 1997 to 3.7 EU/ml in 2007 (p < 0.001). Corresponding figures for adults were less reduced from 12.8 EU/ml in 1997 to 8.3 EU/ml in 2007 (p < 0.001).

As all recently vaccinated children were excluded before the comparisons and as pertussis vaccinations of the adults were administered many years ago, we can note that:

- Children and adults in 1997 had very similar reverse cumulative anti-PT distributions, i.e. they have been exposed to the pertussis agents in the same manner,
- Children in 2007 are more affected by the new pertussis childhood vaccination program than are adults in 2007. However, vaccination of children also has effect on transmission in older age groups.

#### Anti-PT concentrations by age

In a next step, ‘not recently vaccinated’ people sampled in 1997 and 2007 were organized in eight comparable age groups, four for children and four for adults 20 years or older; the age was decided by the time the blood was sampled. The distributions of anti-PT concentrations of 3011 sera from 1997 were compared with comparable 1791 sera collected in 2007 (bold figures in Table 1).

#### Children

**Fig. 2:**
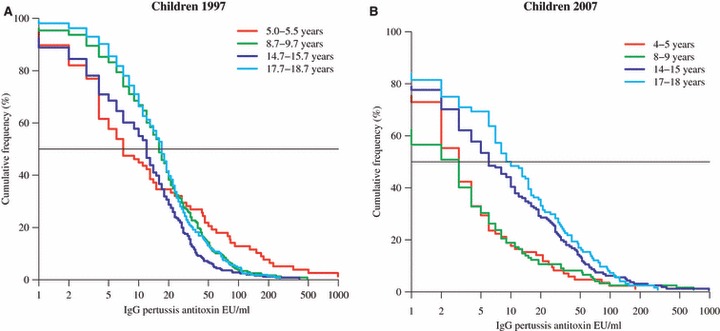
(A, B) Sero-survey 1997 and 2007. Reverse cumulative distribution curves of ELISA IgG anti-PT antibodies by comparable age groups in children after exclusion of ‘recently’ vaccinated children.

In both 1997 and 2007, the highest proportion of detectable anti-PT concentrations (≥1 EU/ml) was observed in the oldest age groups 17.7–18.7 years in 1997 and 17–18 years in 2007 (99% and 83%, respectively), although the proportion was significantly lower in 2007 (p < 0.001), a decade after the reintroduction of pertussis vaccination (Fig. 2A,B). In 1997, at least 90% of the other age cohorts had measurable levels of anti-PT (Fig. 2A), probably because of continuous exposure.

In 2007, the situation had partly changed (Fig. 2B). The proportion of sero-positives was the lowest among children 8–9 years of age, just below 60%, and in the group aged 4–5 years, somewhat higher, but still well below 80%. These two groups were primovaccinated, but not yet given a booster.

Interestingly, the 5.0–5.5 year curve in 1997 and the 4–5 and 8–9 year curves in 2007 (Fig. 2A,B) show the lowest median values.

#### Adult

**Fig. 3:**
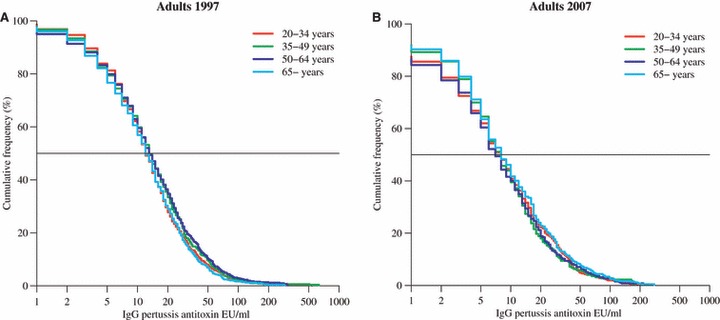
(A, B) Sero-survey 1997 and 2007. Reverse cumulative distribution curves of ELISA IgG anti-PT antibodies in adults by comparable age groups. No cohort was vaccinated after 1979.

For adults, the antibody profile was largely the same when the four age groups were compared with one another in 1997 and 2007 (Fig. 3A,B). The proportion of sera without measurable anti-PT concentrations (<1 EU/ml), on the other hand, was 2.4% in the material from 1997 reflecting the antigenic pressure at the end of the 17-year period without general pertussis vaccination. In 2007, there was a higher proportion, 11.0% (p < 0.001) of sera without measurable anti-PT concentration than in 1997, varying with age group.

#### Anti-PT concentrations <1 EU/ml by age in 1997 and 2007

The proportion of sera (for all people sampled in comparable age groups and after exclusion of “recently vaccinated”) without measurable anti-PT increased significantly from 3.8% in 1997 to 16.3% in people sampled in 2007 (p = 0.002).

**Fig. 4:**
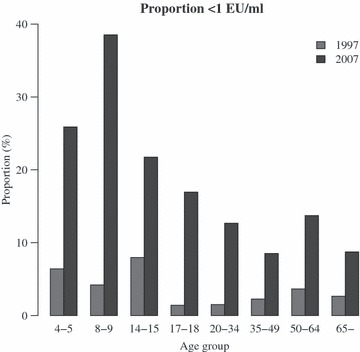
Sero-survey 1997 and 2007. The proportion of sera with concentrations below MLD (minimum level of detection) distributed by age groups after exclusion of ‘recently’ vaccinated children and non-comparable age groups.

Comparing age groups in 1997, the proportions of seronegative were the highest for the three youngest age groups, 5–8%, and generally lower among adults, 2–4%. The highest proportion of seronegatives in 2007, almost 40%, was found in the 8–9 year cohort and the proportion without measurable anti-PT antibodies was continuously falling to about 10% in the group 35-years of age. In each comparable age group, the proportion of seronegatives was considerably higher in 2007 compared with that in 1997 (Fig. 4).

In both materials, cord blood was included to analyze the immune status of pregnant women. In 1997, two of 200 sera from pregnant women (1%) were below MLD. In 2007, there were 377 such sera and 46 of them (12%) had anti-PT concentrations below MLD (p =< 0.001).

#### Seroprevalence of anti-PT ≥50 and ≥100 EU/ml by age in 1997 and 2007

The distributions by age of anti-PT concentrations above two cutoff points, 50 and 100 EU/ml, were examined and compared using 3011 sera from 1997 and 1791 comparable sera from 2007. A sample with an anti-PT concentration above a cutoff point is named a ‘positive’ sample, and the percentage of positive samples for a group of people is named the ‘sero-prevalence’ for the actual group. (We sometimes use the term ‘recent infection’ for a positive sample.)

**Fig. 5:**
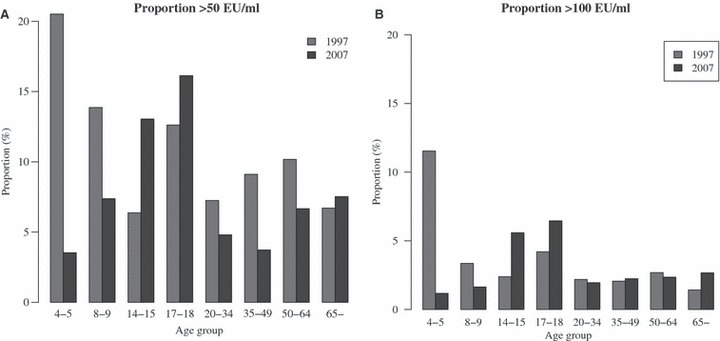
(A, B) Sero-survey 1997 and 2007. The proportion of sera with concentrations ≥50 and ≥100 EU/ml, respectively, distributed by age groups after exclusion of ‘recently’ vaccinated children and non-comparable age groups.

Sero-prevalence, at both cutoff points, shows clear, but somewhat different trends by age for sera collected in both 1997 and 2007 (Fig. 5A,B). In the three youngest age groups sampled in 1997, there was a downward trend in sero-prevalence, at both cutoff points – 21% for 5.0- to 5.5-year-old children down to 7% for 14.7- to 15.7-year-old children at the lower cutoff (p = 0.053 in a test for trend between the four age groups).

In the four child groups sampled in 2007, there was, on the contrary, an opposite upward trend of the sero-prevalence at both cut off points – from 4% for 4–5 year old children to 16% for 17–18 year old at the lower cutoff (p = 0.0012 in a test for trend among the four age groups).

The two diverging trends among children sampled in 1997 and 2007 can be summarized as follows. For younger children, primo-vaccinated in the 2007 samples, but not in the 1997 ones, the proportion sero-positives was significantly lower in 2007 than in 1997 (p = 0.0006 at 50 EU/ml and p = 0.014 at 100 EU/ml). For the two non primo-vaccinated age groups, 14–18 years of age, the proportion of children above the cutoff point was higher in the 2007 samples for both 50 (p = 0.046) and 100 EU/ml (p = 0.11).

In the three adult groups 20–65 years of age, there was a lower sero-prevalence in 2007 than in 1997 at 50 EU/ml (7–10% in 1997 against 4–8% in 2007 with p = 0.052). There was also a trend of increasing proportions of positive samples by age in 1997. At 100 EU/ml, only minor and insignificant differences in sero-prevalence were observed between the four adult age groups and between the two sampled years.

The proportion of sera with high concentrations (≥100 EU/ml), possibly indicating a recent pertussis infection, was about 3% in both materials.

## Discussion

In addition to continuous surveillance programs based on notification systems, serosurveys may be useful for follow-up of pertussis epidemiology. We had the opportunity to study and compare two such materials in Sweden, one sampled in 1997 one year after organized vaccination of infants against pertussis had been reintroduced in 1996 after 17 years without general vaccination, and another sampled 10 years later. The anti-PT assay was carefully monitored for imprecision and drift over time as described under Material and Methods.

Changes in ELISA IgG anti-PT profiles, in 2007 compared with 1997, might indicate an effect of vaccination on the circulation and transmission of pertussis infection. For this comparison, we first excluded children in the sero samples who had been ‘recently vaccinated’, defined as vaccinations that had occurred at least within 2 years before the serum sample was taken. The anti-PT decay curve after vaccination of young infants was far below concentrations of 100 EU/ml already 3 months after primary vaccination and below 50 EU/ml after 6 months (18).

Thereafter, comparisons of ‘ongoing or recent infection’ in comparable age groups were made at ≥50 and ≥100 EU/ml. In infected children not primed with pertussis toxoid, all anti-PT concentrations were below 100 EU/ml after 18 months, but 23% still had levels of ≥50 at that time (18). Unfortunately, we had no data available describing the shape of the antibody decay curves at other ages or after booster vaccinations.

The results indicate a reduced exposure. The proportion of sera without measurable anti-PT for all ages increased significantly from 3.8% to 16.3%. For cord blood sample, this proportion increased significantly from 1% to 12% between 1997 and 2007.

Cord blood samples were included in the age groups of the mothers. The concentration of ELISA IgG anti-PT has been reported to be higher in cord blood than in maternal delivery serum (24). The cord blood results were therefore compared with a control group of women of corresponding ages. As described under Statistics, there were, however, no significant differences in the present material.

For the whole age group of 20 years and more, the reduction in samples with anti-PT concentrations ≥50 EU/ml between 1997 and 2007 was close to statistically significant.

According to the decay curve for infected children not primed with pertussis toxoid, an anti-PT concentration ≥50 EU/ml could mean an infection within the two last years. Thus, evidently unrecognized pertussis infections are still circulating in the grown up population.

Interestingly, there is a successive increase of sero-prevalence by age both in the 1997 and the 2007 materials, although on a lower level in 2007, but for those aged above 65, the proportion above both cutoff points was actually higher in 2007 than in 1997. This may indicate waning immunity possibly in combination with renewed exposure to the youngest generations.

Taken together, the *higher* proportion of sera with undetectable concentrations of anti-PT and the *lower* frequency of infections for adults 2007 compared with 1997 indicate a risk of reduced herd immunity for these age groups in the future. A consequence may be that adult pertussis becomes a problem also in Sweden as it already has been reported in other countries.

For children and adolescents, there are different scenarios of ‘recent infection’. The 1997 samples from children 5.0–5.5 and 8.7–9.7 years of age, born before 1994 and unvaccinated, showed significantly higher sero-prevalence than samples from comparable and not recently vaccinated age groups in 2007, reflecting an effect of the vaccination program against pertussis introduced in 1996. In contrast, those aged 14–18 years had lower sero-prevalence at both cutoff levels in 1997 than in 2007, although statistically significant only for ≥50 EU/ml.

Trend tests on all age groups younger than 20 years showed a close-to-significant *decrease* between 5 and 15 years of age in the 1997 cohort. In contrast, a significant *increase* from 4 to 18 years of age at ≥50 EU/ml was seen in 2007.

Of specific interest is the upward trend by age of ‘recent infections’ in 2007 starting already from 4 years of age and up to 18 years. It may be that the vaccination program has a good initial, but temporary effect on the population immunity. In the unvaccinated society, the waning of immunity starts later, either because of a more long-lasting immunity after natural infection or because of continuous boosting during childhood.

The estimates of ‘recent’ pertussis infections in the population achieved in this study are partly in agreement with some other studies. Using a high titer threshold as indicative of recent natural infection, Nardone et al. (25) reported an age-adjusted prevalence of pertussis infection of 1.2%, higher in children than in adults. In another study using the same cutoff, the sero-prevalence was analyzed in six European countries (14). Recent infection was found to be significantly more likely in adolescents (10–19 years old) and adults in high-coverage countries, whereas infection was more likely in children (3–9 years old) than in adolescents in low-coverage countries (<90%).

Cherry followed up 51 health care workers with serology in the United States and found an average annual rate of 8% based on anti-PT (9). In contrast, Wright et al. found annual incidence rates of 1.3% and 3.6% among physicians in training and among emergency department staff (26).

Our next step is to use the present material to form a basis of mathematical models for estimation of annual seroincidences. Other groups have made such attempts. In a review by von Konig et al., it was concluded that serological responses to *B. pertussis* antigens occur in the general population at a rate between 1.0% and 5% per year depending on the definition (5).

From the Netherlands, de Melker et al. (27) reported an overall yearly incidence of infection of 6.6% for persons aged 3–79 years. The incidence of notified cases was 0.01%. They calculated ELISA antibody cutoff values to PT on the basis of antibody decay curves of patients with pertussis.

Rohani et al., using high-resolution pertussis notification data, reported that vaccination has substantially reduced transmission of pertussis in England and Wales (28).

The present results can be seen as an indication that childhood vaccination against pertussis has slowed down the transmission of pertussis in the Swedish population with a possible negative effect on the population immunity. Therefore, the results also support the extension of the vaccination program with pre-school and school leaving boosters, which is in line with international experiences (7). Continuous surveillance of the population for clinical pertussis in different age groups in combination with follow-up with new serosurveys in a 10-year period is important for evaluation of these rather recent changes in infection and transmission.
